# Muscle-driven spinal cord histological and transcriptomic alterations in a myotonic dystrophy mouse model: insights into neuropathy

**DOI:** 10.1093/braincomms/fcaf313

**Published:** 2025-08-25

**Authors:** Guanzhong Shi, Yining Luan, Yuzhen Ouyang, Kangzhi Chen, Kaiyue Zhang, Zeyi Wen, Huan Yang, Kun Huang

**Affiliations:** Department of Neurology, Xiangya Hospital, Central South University, Changsha 410008, China; National Clinical Research Center for Geriatric Disorders, Xiangya Hospital, Central South University, Changsha 410008, China; Xiangya School of Medicine, Central South University, Changsha 410013, China; Department of Neurology, Xiangya Hospital, Central South University, Changsha 410008, China; National Clinical Research Center for Geriatric Disorders, Xiangya Hospital, Central South University, Changsha 410008, China; Xiangya School of Medicine, Central South University, Changsha 410013, China; Department of Neurology, Xiangya Hospital, Central South University, Changsha 410008, China; National Clinical Research Center for Geriatric Disorders, Xiangya Hospital, Central South University, Changsha 410008, China; Xiangya School of Medicine, Central South University, Changsha 410013, China; Department of Neurology, Xiangya Hospital, Central South University, Changsha 410008, China; National Clinical Research Center for Geriatric Disorders, Xiangya Hospital, Central South University, Changsha 410008, China; Xiangya School of Medicine, Central South University, Changsha 410013, China; Department of Neurology, Xiangya Hospital, Central South University, Changsha 410008, China; National Clinical Research Center for Geriatric Disorders, Xiangya Hospital, Central South University, Changsha 410008, China; Xiangya School of Medicine, Central South University, Changsha 410013, China; Department of Neurology, Xiangya Hospital, Central South University, Changsha 410008, China; National Clinical Research Center for Geriatric Disorders, Xiangya Hospital, Central South University, Changsha 410008, China; Department of Neurology, Xiangya Hospital, Central South University, Changsha 410008, China; National Clinical Research Center for Geriatric Disorders, Xiangya Hospital, Central South University, Changsha 410008, China; Department of Neurology, Xiangya Hospital, Central South University, Changsha 410008, China; National Clinical Research Center for Geriatric Disorders, Xiangya Hospital, Central South University, Changsha 410008, China

**Keywords:** myotonic dystrophy, RNA sequencing, spinal cord, alternative splicing, microglia

## Abstract

Myotonic dystrophy type 1 (DM1) is an inherited neuromuscular disorder characterized by muscle weakness, atrophy and myotonia, with multi-system involvement. Recent studies have highlighted the pathological heterogeneity within the CNS of DM1 patients, particularly significant changes in spinal transcriptome expression and alternative splicing. In this study, we conducted a comprehensive transcriptome analysis of the spinal cord in the muscle-specific DM1 mouse model and their wild-type controls across different life stages: young, adult and old age. Our results revealed an age-dependent increase in differential gene expression between DM1 and wild-type mice with a predominance of downregulated genes. Notably, five genes (*Adgre1*, *Ccl3*, *Fcrls*, *Ogfrl1* and *Reg3b*) were consistently differentially expressed across all age groups. We also generated a temporal profile of cell-type proportions and observed reductions in microglia and astrocytes, along with a trend towards increased ventral neuron populations. Additionally, we characterized the temporal splicing alterations in the spinal cord of DM1 mice and compared with homologous exon skipping events in the CNS of DM1 patients. Our RNA sequencing data elucidate the molecular and cellular adaptations of the spinal cord to muscle defects over time, underscoring that splicing abnormalities observed in the CNS of DM1 patients may reflect contributions from muscle pathology. These findings highlight the necessity of a holistic approach to comprehensively understand the complexity of DM1.

## Teaser

The spinal cord of patients with myotonic dystrophy displays more pronounced splicing abnormalities than other nervous tissues. Shi *et al*. reported histological, transcriptional and splicing alterations in the spinal cord of myotonic mice with lesions limited to muscles, providing new insights into the neuro-muscle interdependence and the neuropathy of myotonic dystrophy.

## Graphical Abstract

**Graphical Abstract:**
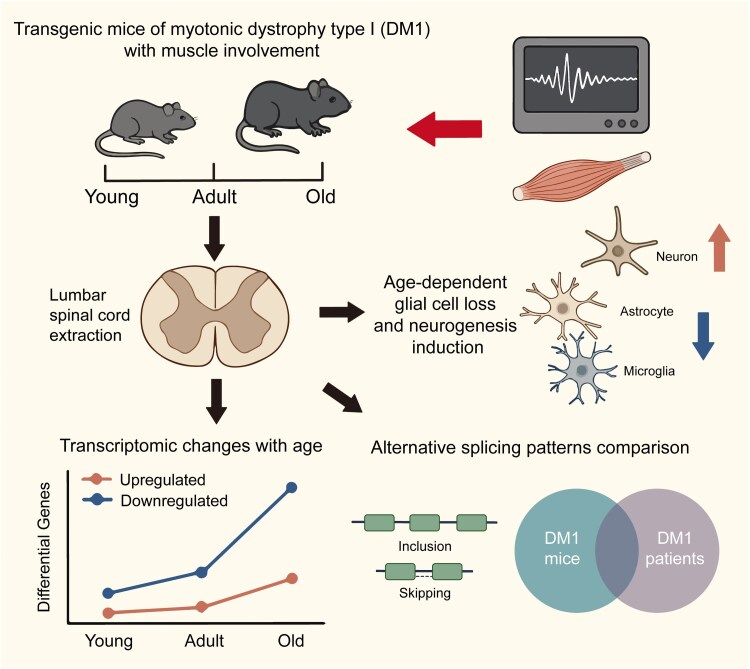

## Introduction

Myotonic dystrophy type 1, also referred to as Steinert’s disease, is the most common form of adult-onset muscular dystrophy, with an estimated global prevalence of ∼9.27 cases per 100 000.^1^ This inherited neuromuscular disorder is characterized by progressive muscle weakness, atrophy and myotonia. It also involves multiple systems, leading to cardiac arrhythmia, cataracts, insulin resistance and CNS alterations.^2^ Myotonic dystrophy type 1 is caused by pathogenic expansion of unstable cytosine-thymine-guanine (CTG) trinucleotide repeats located at the 3′ untranslated region of the dystrophia myotonica protein kinase (*DMPK*) gene on chromosome locus 19q3.3.^3-5^ The *DMPK* gene contains ∼5–37 CTG repeats in healthy individuals, whereas myotonic dystrophy type 1 patients have 50 to thousands of repeats, resulting in a highly variable phenotype that ranges from asymptomatic to severe congenital diseases: the longer CTG repeats are associated with more severe symptoms and earlier onset.^6-8^ Mechanistically, transcripts containing cytosine-uridine-guanine (CUG) repeats form nuclear RNA foci that sequester RNA-binding proteins (RBPs), particularly muscleblind-like (MBNL) proteins, thereby impairing their normal function in RNA processing. Concurrently, pathological overexpression of multiple RBPs, such as CUG-BP and ETR-3-like factors (e.g. CELF1) and HNRNPA1, drives the reversion to foetal splicing patterns observed in adult myotonic dystrophy type 1 tissues.^9-12^ The dysregulation of these RBPs leads to abnormalities in alternative splicing (AS) of many pre-mRNA products and a shift towards foetal splicing patterns in the adult state.^13,14^

Previous studies have observed more apparent abnormalities in the length of CTG repeats and AS in myotonic dystrophy type 1 spinal motor neurons compared to other CNS cells. This highlights a high degree of heterogeneity and complexity in the neuropathological changes associated with myotonic dystrophy type 1.^15,16^ Given the interdependence between muscle and nervous system, it is plausible that muscle defects in myotonic dystrophy type 1 may influence spinal cord function and splicing mechanisms.^17-20^ The HSA^LR^ mouse model, which expresses a human skeletal actin (HSA) gene containing a CTG repeats expansion of ∼220 repeats, is well established for studying myotonic dystrophy type 1. This model specifically induces pathological changes in skeletal muscles, resulting in classic myotonic phenotype and characteristic pathological patterns in muscle tissue, but without muscle atrophy or weakness.^21,22^ Although this limitation restricts the use of HSA^LR^ mice in studying systemic effects beyond muscles in myotonic dystrophy type 1, it provides a unique opportunity to explore the independent effects of myotonic dystrophy type 1 muscle defects on the nervous system.

In this study, we performed RNA sequencing (RNA-seq) to investigate transcriptome and splicing alterations in the spinal cord of HSA^LR^ mice. We compared these findings with existing data from myotonic dystrophy type 1 patients. We aimed to explore the potential effects of muscle pathology on the transcriptional level of the spinal cord and elucidate whether muscle defects contribute to the splicing abnormalities observed in the CNS of myotonic dystrophy type 1 patients. This comprehensive approach will enhance our understanding of the systemic nature of myotonic dystrophy type 1 and may uncover novel therapeutic targets for mitigating its neurological effects.

## Materials and methods

### Animals

The Department of Experimental Animals at Central South University authorized all mouse research, ensuring adherence to relevant ethical guidelines. HSA^LR^ transgenic mice (strain number: 032031) were obtained from Jackson Laboratory in the USA. These mice express over 220 untranslated CUG repeat sequences driven by muscle-specific promoters.^21^ Wild-type (WT) FVB/N mice with an identical genetic background were acquired from the Experimental Animal Department of Central South University.

### Spinal cord isolation and RNA extraction

HSA^LR^ and WT mice were divided into young, adult and old groups and terminated using carbon dioxide asphyxiation instead of cervical dislocation at 35 (young), 165 (adult) and 456 (old) days old to prevent any damage to the spinal cord. Immerse mice in a solution of 70% ethanol and eliminate the fur from their dorsal region. Make a midline incision from the head to the tail, and then carefully remove the skin and muscle layers to expose the vertebral column. Cut through the vertebral column along the midline and gently separate the vertebrae to reveal the spinal cord. Carefully lift the spinal cord from the lumbar region using fine forceps, ensuring minimal tissue damage. Following the manufacturer’s instructions, we isolated total RNA from spinal cord tissues using TRIzol reagent (Invitrogen). Evaluate RNA purity and quantity using a NanoDrop 2000 spectrophotometer (Thermo Scientific). Use the Agilent 2100 Bioanalyzer (Agilent Technologies) to test RNA integrity. Generally, acquired RNA samples consistently have an RNA integrity number of 8.5 or higher. Then, following the manufacturer’s instructions, use the VAHTS Universal V6 RNA-seq library preparation kit to construct the library.

### RNA library construction and sequencing

The libraries were sequenced using the Illumina NovaSeq 6000 platform, producing paired-end reads of 150 bp. For each sample, around 49.40 million raw reads were obtained. Initially, the raw data were processed in FASTQ format using Fastp to remove low-quality reads. This produced 48.38 million clean reads per sample for further analysis. HISAT2 was subsequently utilized to map these clean reads to the reference genome. Gene expression was measured in fragments per kilobase of transcript per million mapped reads (FPKM) and quantified using HTSeq-count. This normalization method accounts for variations in library size and gene length.

### Quantitative reverse transcription PCR validation

RNA extraction from the spinal cord of mice was performed as described previously with minor modifications.^23,24^ The spinal cord was homogenized using a high-speed tissue homogenizer (Servicebio, KZ-II) in the lysis buffer from the FastPure Cell/Tissue Total RNA Isolation Kit (Vazyme, Cat. RC101-01). Total RNA from the spinal cord was extracted by the kit according to the manufacturer’s instructions. RNA was reverse transcribed into cDNA using RevertAid Master Mix (Thermo Fisher, Cat. M1631). Quantitative reverse transcription PCR (qRT-PCR) was performed with the QuantStudio (Thermo Fisher Scientific) using the NovoStart® SYBR qPCR SuperMix Plus (Novoprotein, Cat. E096). Gene expression levels were normalized to that of glyceraldehyde-3-phosphate dehydrogenase (*Gapdh*). All qRT-PCR experiments were performed in duplicate.

### Immunofluorescence

Following transcardial perfusion with 4% paraformaldehyde (PFA), spinal cords were carefully dissected from adult mice, cryoprotected in 30% sucrose and embedded in optimal cutting temperature (OCT) compound. Serial 20-μm-thick coronal sections were obtained using a cryostat. Adjacent sections were systematically distributed into three independent staining sets for the specific detection of astrocytes (glial fibrillary acidic protein, GFAP), microglia (Iba1) and neurons (NeuN), respectively. All sections were blocked in PBS containing 5% normal serum and 0.3% Triton X-100 to reduce non-specific binding. Primary antibodies against GFAP (1:800, Abcam #ab7260), Iba1 (1:500, Abcam #ab178846) and NeuN (1:200, Abcam #ab177487) were applied to their corresponding section sets and incubated at 4°C overnight (∼24 h). Following thorough washes, sections were incubated with species-specific fluorescent secondary antibodies and counterstained with 4′,6-diamidino-2-phenylindole to visualize nuclei. Fluorescence intensity was quantified using ZEN software (version 3.6, Carl Zeiss) to ensure consistent and objective measurement across samples.

### Haematoxylin-eosin (HE) staining

Following euthanasia under deep isoflurane anaesthesia, bilateral tibialis anterior muscles were rapidly dissected from mice. Muscle specimens were gently isolated along the myofibre orientation, rinsed in ice-cold saline to remove residual blood and embedded in OCT compound within 60 s post-harvest. Tissues were flash frozen in liquid nitrogen-chilled isopentane (−160°C, 90 s) and stored at −80°C until processing. For histopathological analysis, frozen muscles were fixed in 4% PFA for 5 min, followed by sequential staining: immersion in Harris haematoxylin (6 min), differentiation in 0.5% acid ethanol (5 s), bluing in running tap water (10 min), counterstaining with 0.5% eosin Y (3 min), dehydration through graded ethanol series and xylene clearing. Sections were coverslipped with neutral resin and dried at 37°C.

### Statistical analyses

All statistical analyses were performed using R (v4.4.0), with specific packages and parameters detailed below. The code used for the analyses is provided as an R Markdown (Code.Rmd) file in the Supplementary material. Experimental units for animal studies were defined as individual mice. For RNA-seq data, raw counts were processed using DESeq2 (v1.44.0) to identify differentially expressed genes (DEGs) with a Wald test, applying a false discovery rate (FDR) correction for multiple comparisons (Benjamini–Hochberg method).^25^ DEGs were defined using thresholds of |log_2_ fold change (FC)| > 1 and FDR-adjusted *P* < 0.05. DecoupleR (v2.9.7) was used to perform a prior knowledge analysis on the calculated DEGs, and linear regression was used to determine the activity of several important transcription factors (TFs) and pathways.^26^ For temporal gene expression profiling, maSigPro (v1.76.0) implemented a quadratic regression model with FDR < 0.05 to cluster genes into dynamic expression patterns. Gene Ontology (GO) enrichment analysis was performed using clusterProfiler (v4.12.2), with Bonferroni adjusted *P* < 0.05 as significance criteria.^27^ We used BayesPrism (v2.2.2) to simulate the cell-type-specific expression profile in mouse spinal cord based on the single-cell RNA-seq (scRNA-seq) results of Matson *et al*. and estimated the posterior distribution and cell-type composition of cell-type-specific gene expression based on the expression of bulk RNA-seq in our samples.^28,29^

To identify differential AS events across various samples, we utilized replicate multivariate analysis of transcript splicing (rMATS v4.0.2).^30^ Initially, reads were aligned to the *Mus musculus* reference genome using TopHat v2.0.13 with default parameters, allowing for up to two mismatches. WT mice served as the control group for AS analysis using rMATS. Five types of AS events were identified: mutually exclusive exons, retained introns, alternative 5′ splice sites, alternative 3′ splice sites and skipped exons (SEs). Differential AS events were determined using an FDR of <0.05 and a |differential percent spliced-in (dPSI)| of >0.1. The |dPSI| > 0.1 threshold was selected based on prior studies to balance statistical significance, biological relevance and false-positive control for low-effect-size events.^15,31^

For immunofluorescence, reverse transcription-PCR (RT-PCR) and qRT-PCR validation, data were analysed using two-tailed Student’s *t*-tests (α = 0.05) for two-group comparisons (e.g. HSA^LR^ versus WT), with exact test statistics (*t* value) and *P*-values reported in figure legends.

## Results

### Age-dependent differential gene expression and cellular composition in HSA^LR^ mice

**Figure 1:**
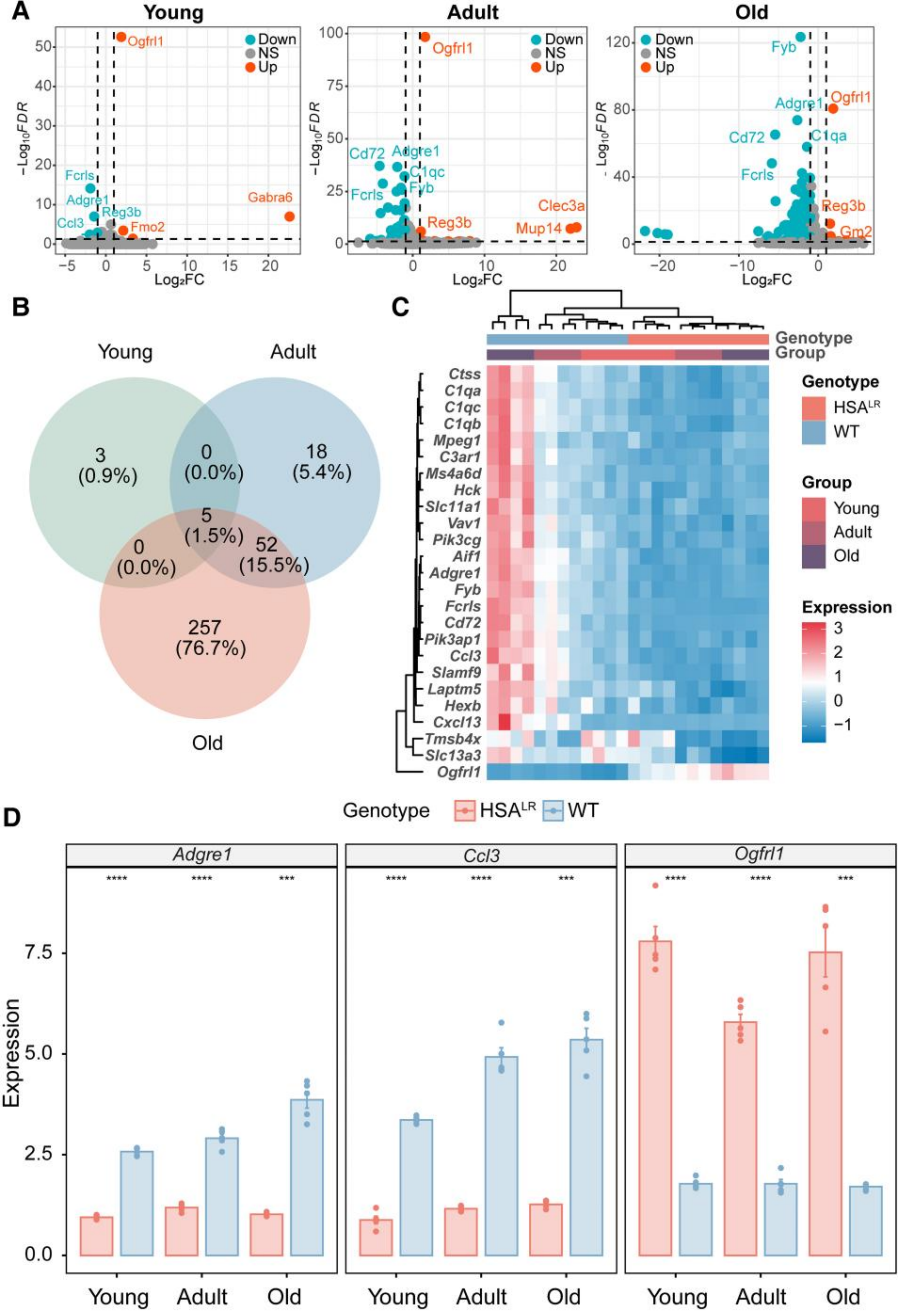
**Differential gene expression analysis in the spinal cord of HSA^LR^ mice.** (**A**) Volcano plots display significant DEGs, identified using DESeq2 with the Wald test. Genes were considered significant if they met the criteria of an FDR < 0.05 and an |log_2_FC| > 1. Each group consisted of *n* = 4 mice. Orange dots indicate upregulated genes, green dots represent downregulated genes and grey dots correspond to NS-regulated genes. (**B**) Venn diagrams illustrating the overlap of DEGs across three age groups. (**C**) Heatmap displaying the top 25 DEGs in the aged mouse group. Coloured bars above the heatmap indicate mouse age and genotype (*N* = 4 mice per group; total *N* = 24). Each row represents *Z*-score–transformed log_2_(1 + FPKM) expression values across all samples, with blue indicating lower expression and red indicating higher expression. (**D**) qRT-PCR validation of three robust DEGs (*Ccl3*, *Adgre1*, *Ogfrl1*) in the spinal cord of HSA^LR^ mice. Individual data points correspond to biological replicates (*N* = 5 mice/group; Student’s *t*-test). Data are shown as means ± standard deviation. Significant differences (****P  *<* 0.0001; ***P  *<* 0.001; **P  *<* 0.01; *P  *<* 0.05) were observed for *Ogfrl1* (young, *t* = −16.14, *P* = 6.26e-05; adult, *t* = −17.98, *P* = 1.42e-06; old *t* = −9.528, *P* = 6.56–04), *Ccl3* (young, *t* = 24.78, *P* = 1.38e-06; adult, *t* = 16.6, *P* = 6.47e-05; old *t* = 14.36, *P* = 1.07e-04) and *Adgre1* (young, *t* = 38.31, *P* = 1.24e-08; adult, *t* = 16.15, *P* = 9.19e-06; old *t* = 13.65, *P* = 1.50e-04) across all age groups. Red bars represent HSA^LR^ samples, and blue bars represent WT samples. DEG, differentially expressed genes; FDR, false discovery rate; log_2_FC, log_2_ fold change; NS, non-significant; qRT-PCR, quantitative reverse transcription PCR; HSA^LR^, transgenic mice with a human skeletal α-actin gene modified by the insertion of 220 CTG repeats; WT, wild-type.

In this study, we analysed 24 spinal cord samples from male HSA^LR^ mice, divided into young, adult and old groups (four samples each), along with age-matched WT mice. Using DESeq2 for differential gene expression analysis, we identified 374 significant DEGs (FDR < 0.05, |log_2_FC| > 1), predominantly downregulated. The volcano plot (Fig. 1A) revealed the greatest expression differences in the old group, with 314 DEGs—seven upregulated and 307 downregulated. In the young group, eight genes exhibited significant differential expression, equally split between upregulated and downregulated, indicating minimal expression differences. The adult group showed 75 DEGs, with 10 upregulated and 65 downregulated. The Venn diagram (Fig. 1B) highlighted five robust DEGs (*Adgre1*, *Ccl3*, *Fcrls*, *Ogfrl1* and *Reg3b*) consistently differentially expressed across all groups. Notably, most of the DEGs identified in the young group (5/8) are also present in the adult and old groups, with similar patterns observed between the adult and old groups (57/75). The heatmap (Fig. 1C) demonstrated consistent downregulation of *Adgre1*, *Ccl3* and *Fcrls* in all HSA^LR^ groups, while *Ogfrl1* was consistently upregulated. Interestingly, *Reg3b* showed an age-dependent pattern: upregulated in young mice but downregulated in adult and old HSA^LR^ mice. Subsequent qRT-PCR confirmed the relative expression levels of several robust DEGs (Fig. 1D), with *Adgre1* and *Ccl3* levels increasing in HSA^LR^ samples compared to controls, while the opposite trend was observed for *Ogfrl1*.

**Figure 2:**
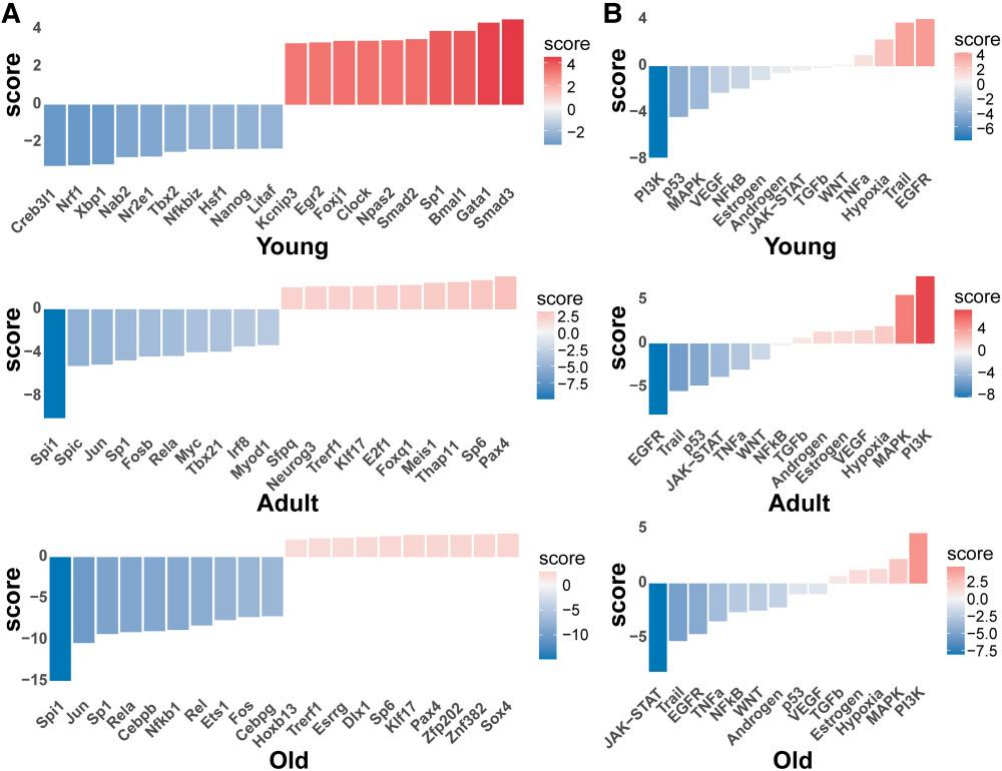
**TF and pathway enrichments.** Major TF (**A**) and pathway (**B**) activities estimated by the DecoupleR algorithm across different groups using bulk RNA-seq expression matrix. Colour scales represent after-scaling expression data.

Based on prior knowledge, we used DecoupleR to infer the activity of major TFs and pathways for gene expression matrices from different groups. Notably, *Spi1* expression exhibited significant downregulation in both the adult and old groups (as depicted in Fig. 2A). In CNS, the expression of *Spi1* is specifically localized to microglia, and its expression level impacts the transcription and activation of microglia.^32,33^ Regarding pathways, we observed that the PI3K pathway was markedly downregulated in the young group but showed elevated expression in the later ages. Furthermore, in both young and old groups, downregulation of the epidermal growth factor receptor (EGFR) and JAK-STAT pathways (Janus Kinase–Signal Transducer and Activator of Transcription pathways) was identified, and the particular cellular gene expression and weighted information in these pathways are displayed in the figure (Figure 2B). Several findings have established that inhibition of EGFR enhances spinal cord injury recovery, whereas the signalling pathway of JAK-STAT plays a crucial role in initiating and modulating innate and adaptive immune responses.

**Figure 3:**
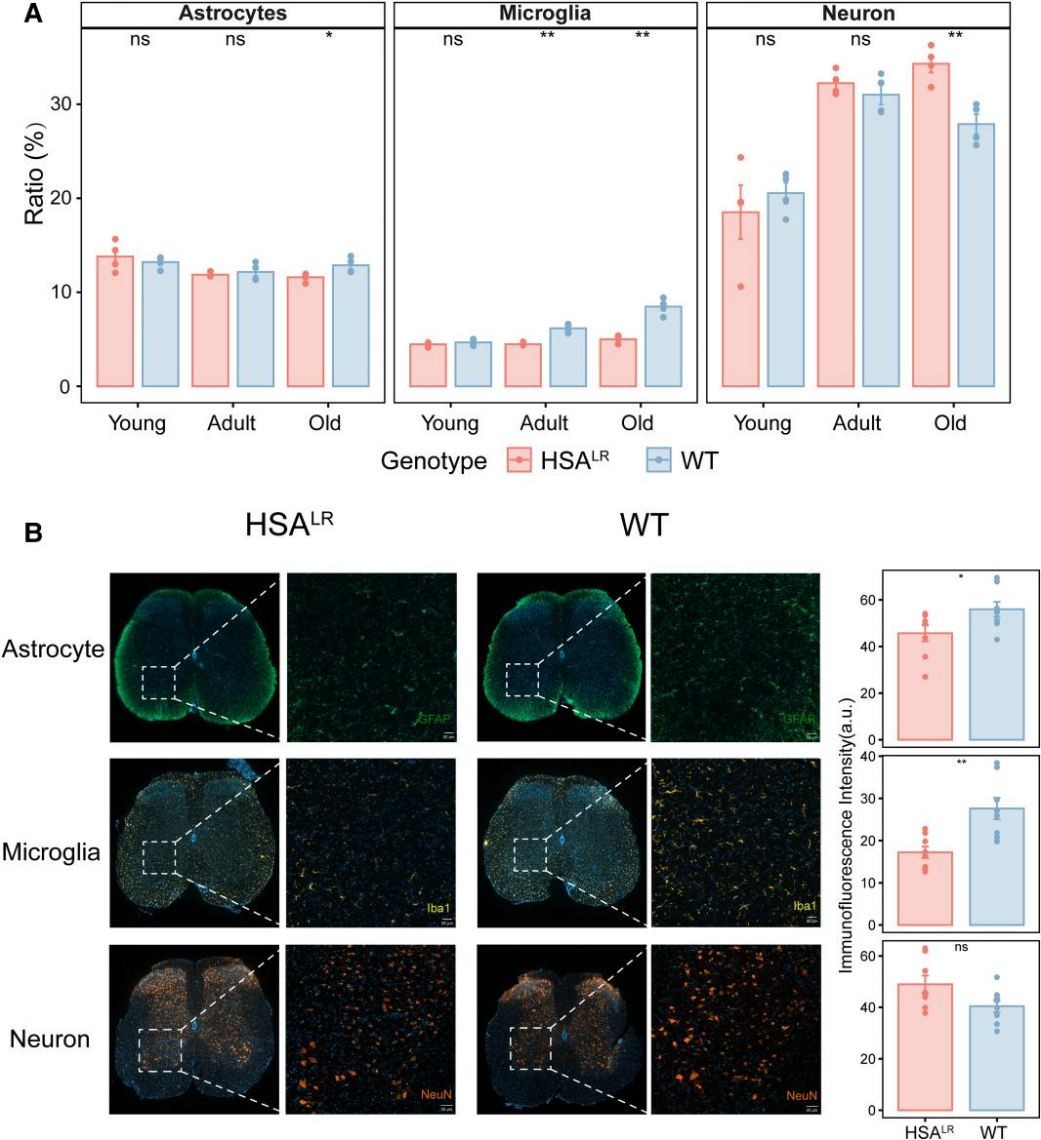
**Relative proportions of major cell types.** (**A**) Boxplot showing the relative proportion of major cell types with significant differences inferred from bulk RNA-seq data using the deconvolution algorithm (*N* = 4 mice/group; Student’s *t*-test). Individual points represent the relative ratio of a cell type in a single sample. Blue boxes/dots, WT samples; red boxes/dots, HSA^LR^ samples. Significant differences (**P  *<* 0.01; *P  *<* 0.05) were observed for microglia in adult (*t* = −7.00; *P* = 0.0026) and old (*t* = −7.26; *P* = 0.0017) stages, neurons (*t* = 4.48; *P* = 0.0044) and astrocytes (*t* = −2.67; *P* = 0.047) in old stage. (**B**) Representative immunofluorescence images of the L1−L2 spinal cord segments from mice in the aged group, showing GFAP^+^ astrocytes (green), Iba1^+^ microglia (yellow) and NeuN^+^ neurons (orange) at 10× magnification. Scale bar: 50 µm. Images were acquired under standardized confocal settings to ensure comparative intensity fidelity across samples. Quantified immunofluorescence intensity at 10× magnification was shown as a bar graph on the right. Histograms compare immunofluorescence intensity per image between WT and HSA^LR^ mice. Individual data points correspond to biological replicates (*N* = 8 mice/group; Student’s *t*-test). Data are shown as means ± standard deviation. Significant differences (**P  *<* 0.01; *P  *<* 0.05) observed in microglia (*t* = −3.3; *P* = 0.0043) and astrocytes (*t* = −2.21; *P* = 0.044) and neurons showed no significant differences (*t* = 2.05; *P* = 0.0619). HSA^LR^, transgenic mice with a human skeletal α-actin gene modified by the insertion of 220 CTG repeats; WT, wild-type.

Subsequently, based on the scRNA-seq results from Matson *et al*. on mouse spinal cord, we used BayesPrism through the deconvolution algorithm to calculate the proportion of each cell type in the spinal cord sample.^29,34^ In adult HSA^LR^ mice, we observed a selective reduction in microglial proportion without significant alterations in other major cell types. However, old HSA^LR^ mice exhibited compounded pathology: astrocytes and microglia were diminished, while total neuronal proportion increased compared to age-matched WT controls (Fig. 3A). Considering the diverse types of neurons within the spinal cord, we subsequently conducted a more detailed inspection of neurons to determine the changing trends of specific types of neurons. As shown in Supplementary Table 1, no significant differences in the number of most types of neurons were observed between HSA^LR^ mice and WT mice, except for a significant increase in the proportion of ventral excitatory neurons in the juvenile group and ventral inhibitory neurons (VINs) in the old group in HSA^LR^ mice. Immunofluorescence staining of specific cell populations (Fig. 3B) revealed a significant reduction in both astrocyte (*P* = 0.044) and microglia (*P* = 0.0043) intensities in aged HSA^LR^ mice. In contrast, ventral neurons exhibited an increased intensity with marginal significance (*P* = 0.062).

To investigate whether this change was caused by the expression of *ACTA1* with (CUG)n repeat expansion in the spinal cord, we verified the expression of *ACTA1* in the spinal cord. As shown in Supplementary Fig. 1, the expression of *ACTA1* in the spinal cord of HSA^LR^ is absent. To further investigate the impact of myotonia on the spinal cord alterations observed in HSA^LR^ mice, we assessed muscle pathology across different age groups. Histological analysis of skeletal muscle tissue revealed distinct pathological alterations, most notably a significant increase in centralized nuclei—a hallmark feature of dystrophic and myopathic conditions (Supplementary Fig. 2). Quantitative analysis showed a significant increase in the proportion of fibres with centralized nuclei in HSA^LR^ mice compared to WT mice in adult and old groups.

### Temporal gene expression profile and functional enrichment in HSA^LR^ mice

**Figure 4:**
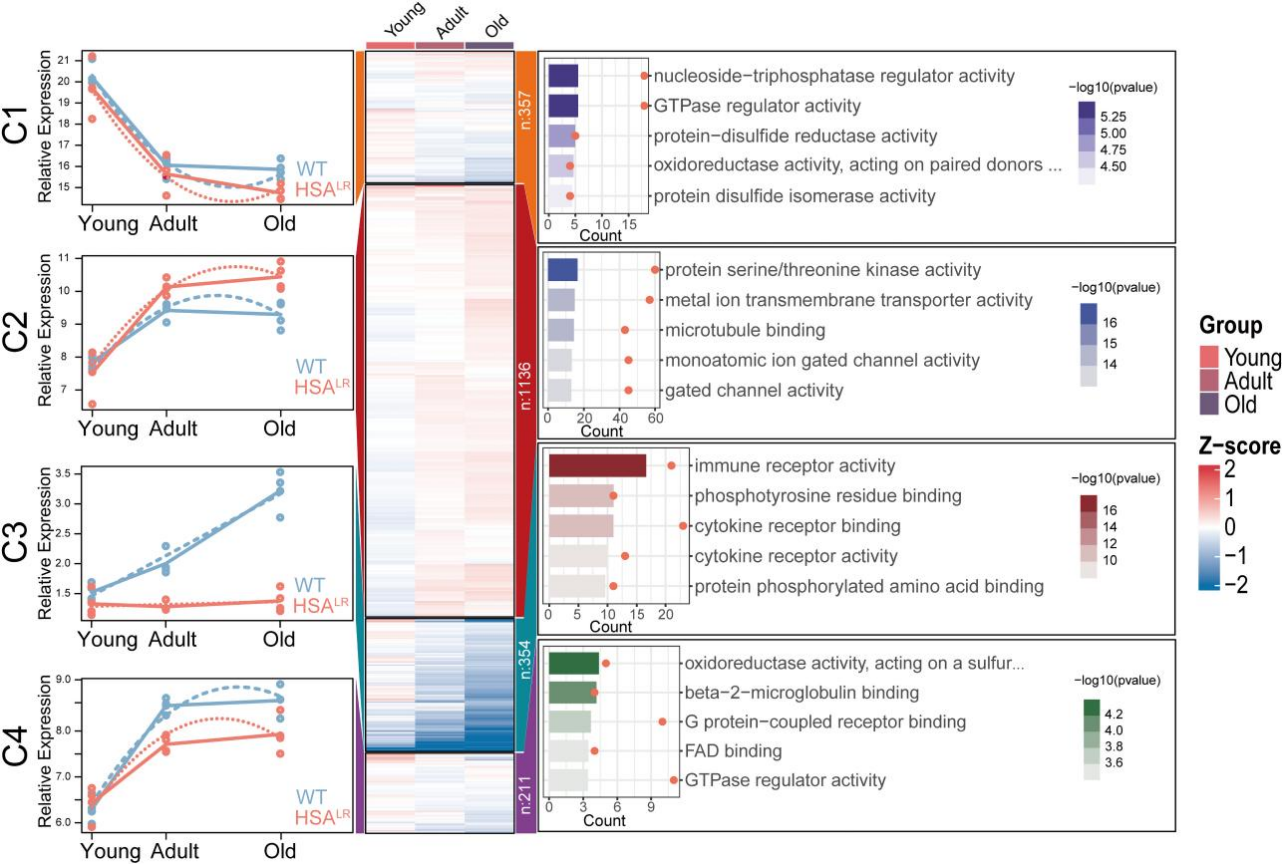
**Clustering and enrichment of genes with different expression profiles over time.** The line graph (left) represents the relative expression values of genes in four clusters in different age groups across different genotype mice, with green representing HSA^LR^ mice and red representing WT mice (*N* = 4 mice/group). The heatmap (middle) displays the log_2_FC values of genes from different clusters, while the top colour block indicates annotations for age groups. The right panel illustrates the top five enriched GO terms for each group; the colour of the bar plot represents the log_10_  *P*-value calculated by hypergeometric test, and the orange dots represent gene count. Gene enrichment was performed via ClusterProfiler (hypergeometric test), which depends on the background gene set (all expressed genes) and the input DEG list. GTP, guanosine triphosphate; HSA^LR^, transgenic mice with a human skeletal α-actin gene modified by the insertion of 220 CTG repeats; WT, wild-type; log_2_FC, log_2_ fold change; GO, Gene Ontology; DEG, differentially expressed genes.

Furthermore, we used maSigPro, which is based on a quadratic regression model, to independently identify genes with different expression patterns between HSA^LR^ and WT mice. This analysis identified 2073 genes whose expression profiles were significantly distinct from the control group (FDR < 0.05). These genes are grouped into four clusters with varied response curves over the entire time span. Increasing the number of clusters to four or more failed to produce any new identifications of distinct expression patterns. Thereafter, to enhance our comprehension of the biological functions and processes associated with genes in each cluster, GO analyses was conducted (Fig. 4).

The first cluster consists of 357 genes whose expression decreases with age, with a more pronounced reduction observed in HSA^LR^ mice. These genes were significantly enriched in activities related to guanosine triphosphatase (GTPase) regulator activity (Bonferroni adjusted *P* = 0.0017), nucleoside triphosphate regulation (Bonferroni adjusted *P* = 0.0017) and protein disulphide reduction (Bonferroni adjusted *P* = 0.0058), indicating a focus on molecular processes involving energy transfer and redox reactions. The second cluster, containing the largest number of genes (*n* = 1136), shows a substantial increase in expression during development, remaining relatively stable or slightly reduced in adulthood in WT mice. However, in HSA^LR^ mice, the expression continues to rise with age. This cluster is significantly associated with pathways related to ion channels and cytoskeletal organization, and it showed enrichment in protein serine/threonine kinase activity (Bonferroni adjusted *P* = 3.26E-17), metal ion transmembrane transporter activity (Bonferroni adjusted *P* = 1.37E-15) and microtubule binding (Bonferroni adjusted *P* = 3.37E-15).

The third cluster comprises 354 genes, whose expression levels progressively increase with age in WT mice while remaining relatively stable in HSA^LR^ animals, with a slight increase observed in the old group. Immune-related pathways, such as immune receptor activity (Bonferroni adjusted *P* = 5.46E-08), phosphotyrosine residue binding (Bonferroni adjusted *P* = 8.18E-08) and cytokine receptor binding (Bonferroni adjusted *P* = 1.58E-06), significantly enrich this gene cluster and align with observed changes in microglia proportions. The fourth cluster, the smallest, shows a continuous increase in gene expression with age, though the increase is less pronounced in HSA^LR^ mice. Genes in this cluster were enriched in oxidoreductase activity, acting on a sulphur group of donors (Bonferroni adjusted *P* = 0.015), beta-2-microglobulin binding (Bonferroni adjusted *P* = 0.015) and G protein–coupled receptor binding (Bonferroni adjusted *P* = 0.027).

### Differential splicing analysis in HSA^LR^ mice and comparison with myotonic dystrophy type 1 patients

**Figure 5:**
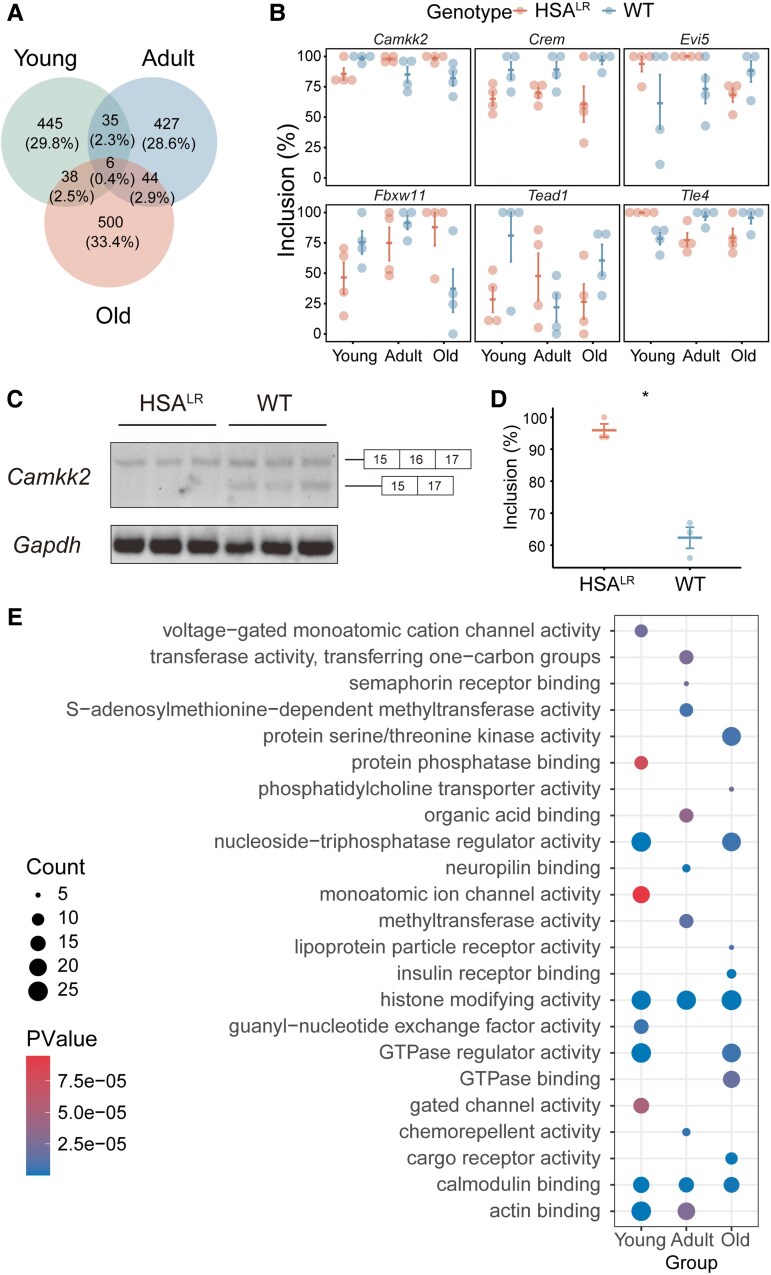
**Age-related overlap and enrichment of SE events.** (**A**) Venn diagram showing the overlap of SE events across different age groups. Colours indicate the specific age group to which each event belongs. (**B**) Inclusion event ratio for significant SE events present in all age groups detected by rMATS. (*N* = 4 mice/group). Colours indicate the specific age group to which each event belongs, and individual points represent biological replicates. **(C)**  *Camkk2* splicing in spinal cord. RT-PCR analysis of *Camkk2* and *Gapdh* in HSA^LR^ and WT spinal cord. Uncropped gels are shown in Supplementary Fig. 3. (**D**) Quantitative analysis of inclusion events of *Camkk2* (*N* = 3 mice/group; Student’s *t*-test). Data are shown as means ± standard deviation. Significant differences (*P  *<* 0.05) were observed for myotonic dystrophy type 1 group with higher inclusion levels (*t* = 8.63; *P* = 0.002) Individual points represent biological replicates. Blue boxes, WT samples; red boxes, HSA^LR^ samples. (**E**) Bubble plot illustrating GO terms enriched with genes associated with SE events in different age groups. The size of each bubble corresponds to the gene count, while the colour gradient indicates the *P*-value calculated by hypergeometric test, with darker colours representing more significant enrichment. HSA^LR^, transgenic mice with a human skeletal α-actin gene modified by the insertion of 220 CTG repeats; WT, wild-type; SE, skipped exon; RT-PCR, reverse transcription-PCR; GO, Gene Ontology.

In light of the numerous transcriptome changes observed in the spinal cord of HSA^LR^ mice, rMATS was utilized to assess variations in AS between HSA^LR^ and WT mice across different age groups (Supplementary Table 2). Extensive differential splicing events were identified in the spinal cords of HSA^LR^ mice. The composition ratio of various differential splicing events was relatively similar across the three groups, with SE events being the most common. Despite screening for significant differential SE events, as shown in Fig. 5A, there was minimal overlap among the groups, with only six SE events common to all. Furthermore, with the exception of decreased inclusion of exon 8 in *Crem* across all ages, these splicing changes were inconsistent within each group, as illustrated in Fig. 5B.

Subsequently, we examined the differential splicing patterns between the spinal cord of adult HSA^LR^ mice and the frontal cortex and spinal cord of myotonic dystrophy type 1 patients. Among the differentially spliced genes in the spinal cord of myotonic dystrophy type 1 patients reported by Nakamori *et al*., 35 homologous genes also exhibited splicing abnormalities in the spinal cord of HSA^LR^ mice, including *Mapt*, *Camkk2*, *Cacna1 g* and *Sorbs1*.^15^ Of the 109 differentially spliced genes in the frontal cortex of myotonic dystrophy type 1 patients identified by Degener, 12 homologous genes were also differentially spliced in the spinal cord of HSA^LR^ mice.^35^ We then humanized the mouse exon segments using LiftOver and compared them with the findings of the aforementioned studies. As illustrated in Supplementary Table 3, Degener *et al*. identified 130 SE events in the frontal lobe of myotonic dystrophy type 1 patients, of which five were also observed in the spinal cord of HSA^LR^ mice*: Sorbs1* exon 17, *Camkk2* exon 16, *Dclk1* exon 17, *Mmy11* exon 42 and *Cacna1g* exon 26. Similarly, Nakamori *et al*. reported SE events in the spinal cord of myotonic dystrophy type 1 patients, with five of these events also present in the spinal cord of HSA^LR^ mice: *Il15ra* exon 3, *Apc* exon 2, *Osbpl8* exon 3, *Camkk2* exon 16 and *Stx3* exon 3. Notably, the only common SE event between the two studies was the skipping of exon 16 in *Camkk2*. Therefore, we examined the AP alterations of *Camkk2* using RT-PCR. Consistent with the RNA-seq results, we observed a reduction of the variant containing exon 16 in HSA^LR^ mice (Fig. 5C and D).

As the bubble plot in Fig. 5E illustrates, the heterogeneity of differential splicing between groups is also reflected in the enriched GO terms related to incorrectly spliced transcripts in each group: genes with differential splicing in young mice are mainly enriched in GTPase regulator activity (Bonferroni adjusted *P* = 4.65E-06), nucleoside-triphosphatase regulator activity (Bonferroni adjusted *P* = 24.65E-06) and calmodulin binding (Bonferroni adjusted *P* = 1.11E-05); adult mice were highly enriched in items such calmodulin binding (Bonferroni adjusted *P* = 3.05E-04), histone-modifying activity (Bonferroni adjusted *P* = 1.77E-03) and chemorepellent activity (Bonferroni adjusted *P* = 3.22E-03). The differentially spliced genes in the elderly group are substantially linked with functions such as insulin receptor binding (Bonferroni adjusted *P* = 3.32E-04), calmodulin binding (Bonferroni adjusted *P* = 5.58E-04) and GTPase regulator activity (Bonferroni adjusted *P* = 1.30E-03).

## Discussion

In this study, we generated and characterized transcriptome and splicing alterations from the spinal cord of HSA^LR^ mice and related controls across different age groups. Our findings revealed adaptive changes in the spinal cord in response to the myotonic dystrophy phenotypes over a longer time scale, and we explored whether these changes are involved in CNS mis-splicings in myotonic dystrophy type 1.

Nerves and muscles are highly interconnected. The influence of nerves on skeletal muscle is particularly evident in cases of persistent neural denervation.^36^ However, muscles reciprocally provide feedback to the nervous system. Skeletal muscles utilize specialized structures such as muscle spindles and Golgi tendon organs to relay information about their state (e.g. changes in length and tension) via sensory fibres to spinal cord interneurons, thereby participating in motor regulation and coordination.^37^ Within spinal cord neural networks, the sensory functions of muscle spindles and Golgi tendon organs are essential for maintaining locomotor precision. Specifically, VINs predominantly receive muscle length signals from Ia afferent fibres of muscle spindles, forming critical inhibitory microcircuits for motor regulation.^38,39^ In the present study, we observed a significant reduction in the proportion of microglia accompanied by an increase in neuronal populations—particularly VINs—within the spinal cord of HSA^LR^ mice. These neurons play a pivotal role in coordinating reflex arcs and motor control, contributing to sensory integration, maintenance of neural circuit homeostasis and fine-tuning of movement.^40,41^ HSA^LR^ mice exhibit a myotonic phenotype marked by sustained muscle hypertonia and delayed relaxation, despite lacking significant skeletal muscle atrophy. This pathological myotonia, a hallmark of the model, may act as a critical driver of the observed spinal cord alterations.

Previous research demonstrates that ageing induces microglial proliferation in the CNS, coupled with a shift towards a pro-inflammatory phenotype marked by upregulated MHC II expression. This age-dependent microglial activation suppresses neurogenesis via inflammatory cytokine release.^42^ Remarkably, exercise as a physiological intervention exhibits potent regulatory effects: voluntary wheel running reverses age-related neurogenesis decline and mitigates cognitive impairment in aged mice.^43,44^ In Alzheimer’s disease transgenic models, treadmill training attenuates age-exacerbated microglial hyperactivation, suggesting exercise-induced neurogenesis is tightly linked to microglial phenotype modulation.^45^ Vukovic *et al*. revealed that exercise-enhanced neurogenesis coincides with reduced MHC II-positive pro-inflammatory microglia, implying exercise may foster a neuroregenerative milieu by curbing microglial overactivation. The CX3CL1–CX3CR1 axis plays a pivotal role: exercise elevates neuronal fractalkine (CX3CL1), which binds microglial CX3CR1 to regulate their functional state.^42^ Concurrently, exercise upregulates neurotrophic factors such as insulin like growth factor 1, synergistically promoting survival and differentiation of neural precursors.^46^ Although exercise reduces activated microglia and enhances pro-neurogenic phenotypes in aged hippocampi, the precise mechanisms governing microglial dynamics remain elusive. Studies on HSA^LR^ mice offer novel insights: despite lacking increased physical activity, their elevated interneuron activity and neurotransmitter release suggest neuronal activity may directly shape microglial behaviour via microenvironmental mediators (e.g. chemokines or inflammatory cytokines).

Additionally, our findings revealed that differential gene expression in the spinal cord of the HSA^LR^ mouse model shows significant overlap across various life stages. This suggests that the persistence and gradual changes in gene expression may be linked to the spinal cord’s adaptive response to muscle defects. The consistent presence of these robust DEGs, specifically *Adgre1*, *Ccl3*, *Fcrls*, *Ogfrl1* and *Reg3b*, throughout different life stages indicates that the spinal cord undergoes sustained molecular regulation to manage muscle defects, highlighting its role in maintaining neuromuscular function and homeostasis. These genes represent a core set of transcriptional changes that persist throughout the mice’s life cycle, underscoring their fundamental role in the potential mechanisms of neuromuscular adaptation and homeostasis. In CNS, *Adgre1* serves as a marker for proliferative microglia.^47^ The *Fcrls* gene is specifically expressed in microglia, distinguishing them from other myeloid cells.^48^  *Ccl3*, also known as macrophage inflammatory protein alpha, is a pro-inflammatory chemokine crucial for CNS inflammation. In neurodegenerative disease specimens, *CCL3* is predominantly expressed by neurons. Its ligands, *CCR3* and *CCR5*, are primarily found in microglia, with elevated levels in reactive microglia.^49^  *CCL3* is highly correlated with the pathological processes of multiple sclerosis and allergic encephalopathy.^50,51^ Long-term injection of *Ccl3* into the lateral ventricle has been shown to inhibit long-term potentiation and impair both spatial and long-term memory in mice.^52^  *Ogfrl1* encodes a protein related to the opioid growth factor receptor. The functional role of *Ogfrl1* gene products remains unclear. However, Oeschger *et al*.^53^ found that *Ogfrl1* is highly expressed in the embryonic sublayer of the cerebral cortex, suggesting its involvement in synapse formation and axonal growth. *In vitro*, neuronal growth on inhibitory substrates was enhanced by *Ogfrl1* transfection.^54^  *Reg3b* is transiently expressed in motor neurons and early sensory neurons during mouse embryonic development. *Reg2*, the equivalent gene of *Reg3b* in rats, acts as a motor neuron survival factor *in vitro*, enhancing axonal repair and regeneration *in vivo*, and is also a potent Schwann cell mitogen.^55^  *Reg3b* knockout mice exhibited delayed myelination of sublingual motor neuron subpopulations, but did not induce motor neuron cell death.^56^

Numerous mis-splicing events have been described in genes expressed in the myotonic dystrophy type 1 brain implicated in its CNS symptomatology, including *MAPT* exons 2–3 and 10, *GRIN1* exons 5 and 21, *APP* exon 8, *MBNL1/2* exon 7 and *CAMK2D* exons 14–15.^16,57-59^ Previous research has revealed that spinal motor neurons in myotonic dystrophy type 1 exhibit more pronounced splicing abnormalities compared to cortical neurons, while the cerebellum exhibits fewer mis-splicing events. This indicates a heterogeneous neuropathy within the CNS.^15^ Currently, the majority of research on myotonic dystrophy type 1 neurological symptoms focuses on cognitive and behavioural problems, whereas only a limited number of papers discuss spinal cord abnormalities in myotonic dystrophy type 1.^60-63^ Nevertheless, the severity of these clinical characteristics or pathological alterations does not consistently correlate with the splicing anomalies. Via LiftOver, we compared the primary homologous SE events in the spinal cord of adult HSA^LR^ mice with those in the nervous system of myotonic dystrophy type 1 patients. Our analysis identified 10 shared SE events between the spinal cord of HSA^LR^ mice and the CNS of myotonic dystrophy type 1 patients, with five of these events showing opposite change (increase or decrease). Currently, there is no research directly linking the genes of five consistent events to neural impairment in myotonic dystrophy type 1. *Myh11* encodes the myosin heavy chain protein and is specifically expressed in smooth muscle cells.^64^ The only available evidence suggests that mutations in *Myh11* are associated with dementia.^65^ AS-mediated functional changes in the Cav3.1/α1G channel of exon 26 of *Cacna1g* may trigger abnormal burst discharges, leading to motor disorders.^66,67^  *Apc* is upregulated in neurons after injury and exerts inhibitory effects on axonal growth through the phosphorylation of β-catenin.^68^ Exon 3 of *Osbpl8* shows increased skipping in the hearts of *Mbnl1/Mbnl2* double knockout mice, but the impact of this event on the nervous system remains unclear.^69^  *Stx3* is associated with docking between synaptic vesicles and presynaptic membranes, and its overexpression leads to increased axonal growth and branching.^70^ Mutations in exon 3 of *Stx3* are associated with cataracts and intellectual disabilities.^71^ These findings suggest that the splicing abnormalities observed in the nervous system of myotonic dystrophy type 1 patients may not be solely due to lesions within the nervous system itself. Instead, they may also reflect contributions from the myotonic dystrophy phenotype. The presence of opposite directional changes in SE events further underscores the complexity of the underlying mechanisms.

In summary, our research underscores the necessity of a holistic approach to comprehensively understand the complex nature of myotonic dystrophy type 1. It is crucial to consider the interactions between various systems under pathological conditions. Future studies are needed to elucidate the specific mechanisms through which myotonic dystrophy type 1 induces transcriptional and splicing abnormalities across different systems. This will enable more targeted and effective therapeutic strategies for myotonic dystrophy type 1.

## Supplementary Material

fcaf313_Supplementary_Data

## Data Availability

The data that support the findings of this study are available from the corresponding author upon reasonable request.
