# Supplementary material for: Muscle-driven spinal cord histological and transcriptomic alterations in a myotonic dystrophy mouse model: insights into neuropathy
Source: Brain Commun. 2025 Aug 25;7(5):fcaf313. doi: 10.1093/braincomms/fcaf313 (PMC12409276; doi:10.1093/braincomms/fcaf313)
Supplement: fcaf313_Supplementary_Data [file fcaf313_supplementary_data.zip › Supplementary_Figures.docx]

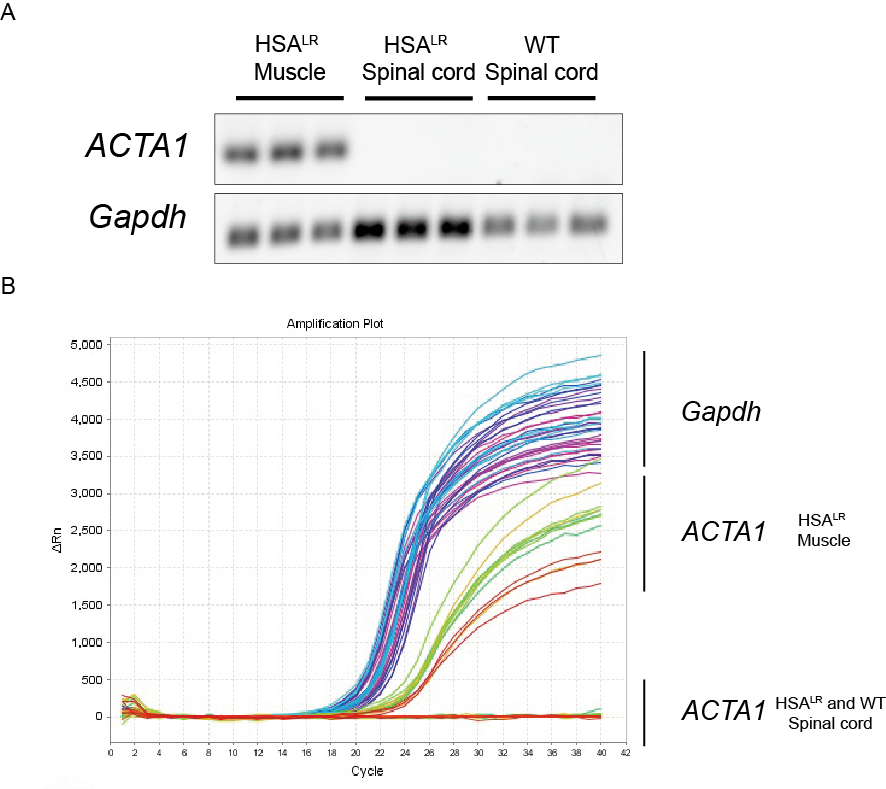


**Supplementary Fig. 1 *ACTA1* expression analysis in spinal cord and muscle. (A)** RT-PCR gel image of *ACTA1* and *Gapdh* in HSA^LR^ and WT mice. (*N* = 3 mice/group) See **Supplementary Figure 3** for uncropped gel images. **(B)** qRT-PCR amplification curves for *ACTA1* in HSA^LR^ spinal cord and muscle, and WT spinal cord normalized to *Gapdh*. (*N* = 3 mice/group) RT-PCR, reverse transcription-PCR ; qRT-PCR, Real-time quantitative reverse transcription PCR; HSA^LR^, transgenic mice with a human skeletal α-actin gene modified by the insertion of 250 CTG repeats; WT, wild-type.


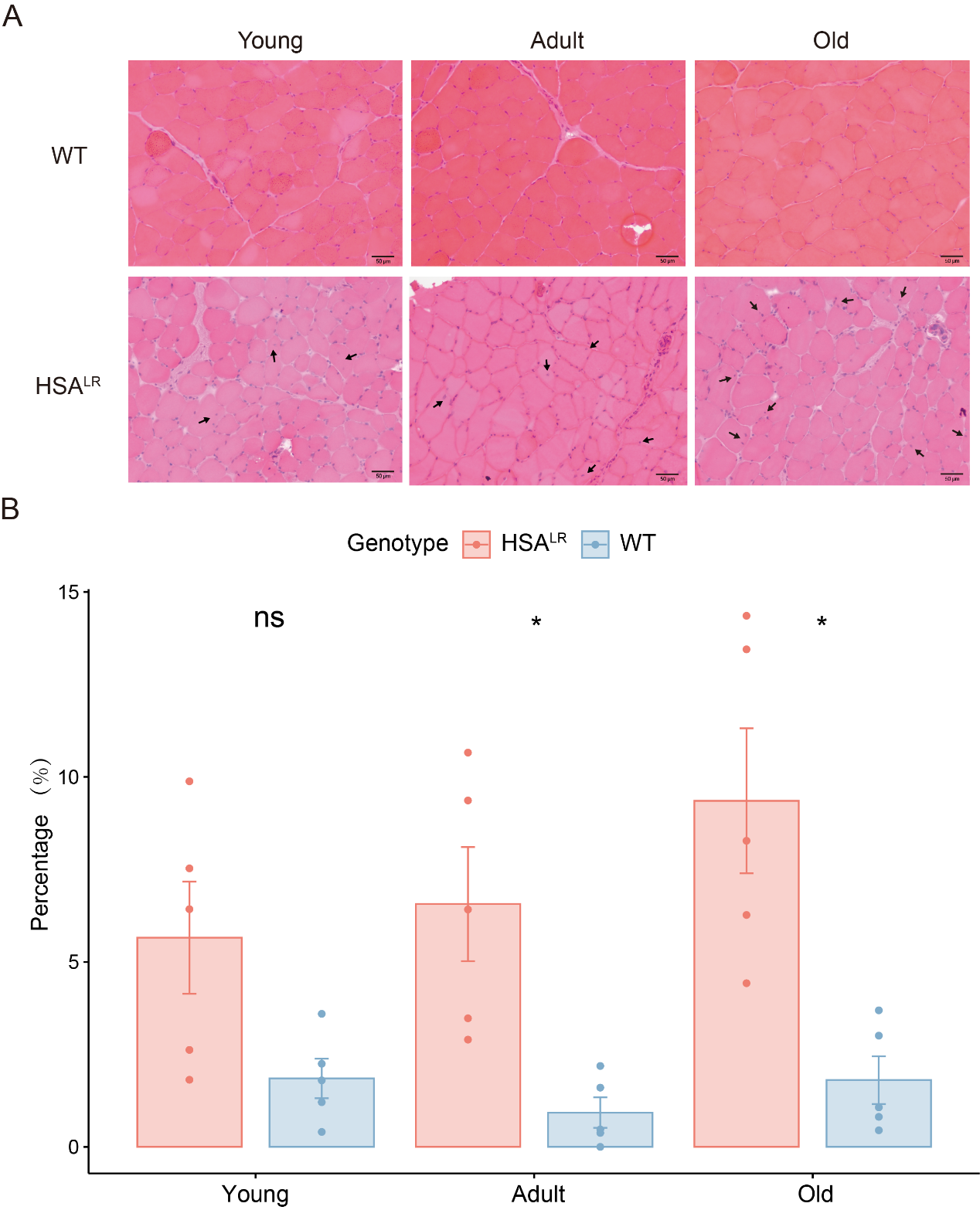


**Supplementary Fig. 2** **Myopathic Changes of HSA^LR^ Mice. (A)** Hematoxylin-eosin staining of TA muscle from HSA^LR^ and WT mice across three age cohorts: young (35 days), adult (165 days), and aged (456 days). Arrows highlight centralized nuclei. Scale bars: 50 μm. **(B)** Quantification of centralized nuclei frequency (percentage of total myonuclei). Individual data points correspond to biological replicates (*N* = 5 mice/group; Student’s t test). Data are shown as means ± standard deviation. Red charts represent HSA^LR^ samples, and blue charts represent WT samples. Significant differences (*p < 0.05) are indicated in adult (*t* = -3.26, *p* = 0.0115) and old (*t* = -3.37, *p* = 0.0103) stages, and young stage showed no significant differences (*p* > 0.05). HSA^LR^, transgenic mice with a human skeletal α-actin gene modified by the insertion of 250 CTG repeats; WT, wild-type; TA, tibialis anterior.

**
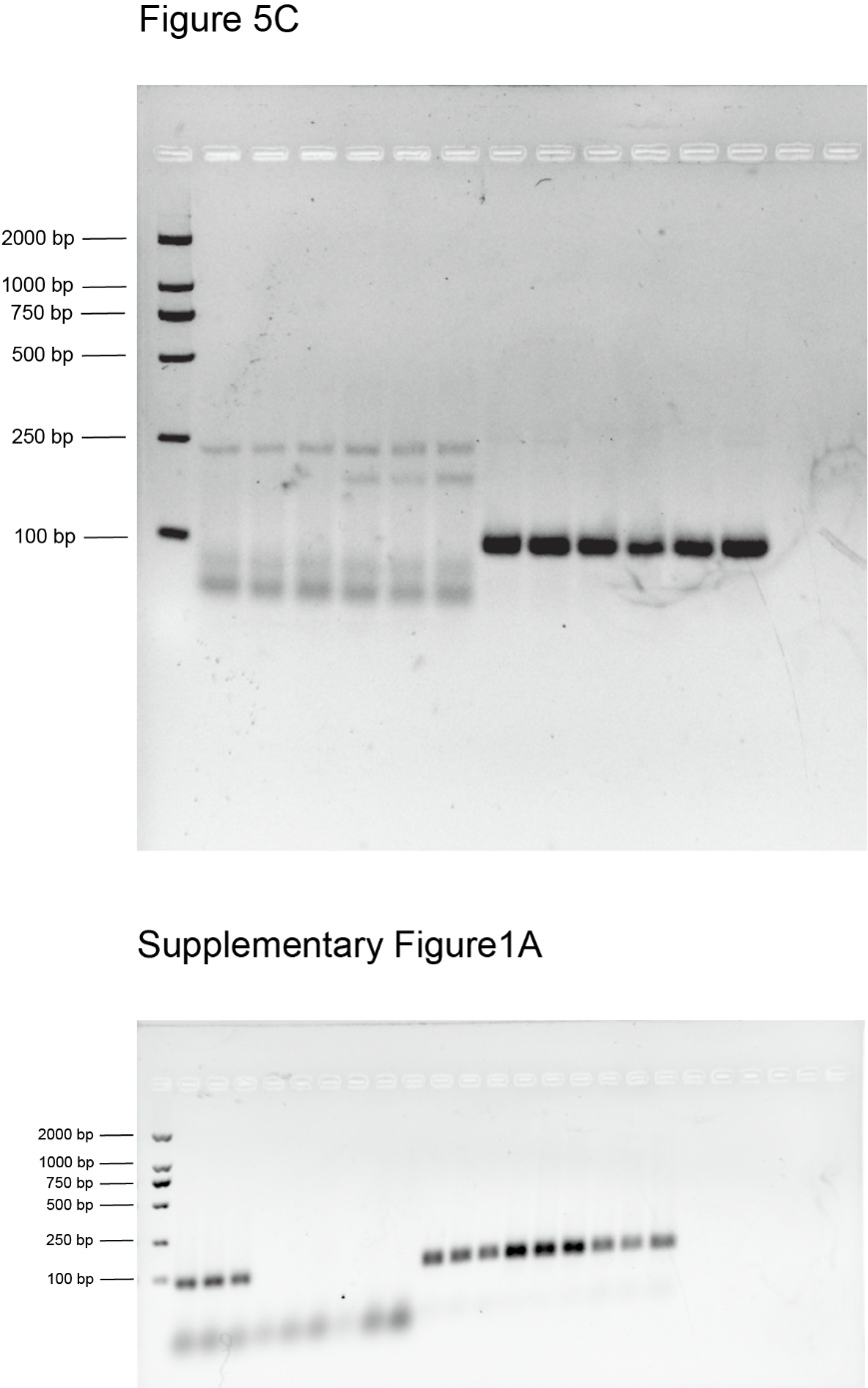
**

**Supplementary Fig. 3 Uncropped gel images of Fig. 5C and Supplementary Fig. 1A.**
